# Genetic polymorphism and natural selection in the C-terminal 42 kDa region of merozoite surface protein-1 (MSP-1) among *Plasmodium knowlesi* samples from Malaysia

**DOI:** 10.1186/s13071-018-3234-5

**Published:** 2018-12-05

**Authors:** Nan Jiun Yap, Indra Vythilingam, Boon Peng Hoh, Xiang Ting Goh, Azdayanti Muslim, Romano Ngui, Yamuna Rajoo, Seow Huey Choy, Timothy William, Tsin Wen Yeo, Yvonne Ai-Lian Lim

**Affiliations:** 1 Department of Parasitology, Faculty of Medicine, University of Malaya, 50603 Kuala Lumpur, Malaysia; 2 Faculty of Medicine & Health Sciences, UCSI University Kuala Lumpur Campus, Cheras, Kuala Lumpur, Malaysia; 3 Department of Medical Microbiology and Parasitology, Faculty of Medicine, Universiti Teknologi MARA (Sungai Buloh Campus), Sungai Buloh, Selangor Malaysia; 4 International Medical University, Bukit Jalil, Kuala Lumpur, Malaysia; 5 Jesselton Medical Centre, 88300 Kota Kinabalu, Sabah Malaysia; 6 Communicable Diseases Centre, Institute of Infectious Disease and Epidemiology, Tan Tock Seng Hospital, Moulmein Road, Singapore, 308433 Singapore; 7 Lee Kong Chian School of Medicine, Nanyang Technological University, 11 Mandalay Road, Singapore, 308232 Singapore; 8 Centre of Excellence for Research in AIDS (CERiA), University of Malaya, 50603 Kuala Lumpur, Malaysia

**Keywords:** *Plasmodium knowlesi*, Malaysia, Merozoite surface protein, Genetic polymorphism, Natural selection

## Abstract

**Background:**

The merozoite surface protein-1 (MSP-1) gene encodes for a leading malaria vaccine candidate antigen. However, its extensive polymorphic nature represents a major obstacle to the development of a protective vaccine. Previously, a pilot study was carried out to explore the sequence variation of the C-terminal 42 kDa fragment within *P. knowlesi* MSP-1 gene (PkMSP-1_42_) based on 12 clinical samples; however, further study on an adequate sample size is vital in estimating the genetic diversity of the parasite population.

**Methods:**

In the present study, we included a larger sample size of *P. knowlesi* (83 samples) covering eight states of Malaysia to determine the genetic polymorphism, natural selection and haplotype groups of the gene fragment coding PkMSP-1_42_. The region flanking PkMSP-1_42_ was amplified by PCR and directly sequenced. Genetic diversity, haplotype diversity, population genetic differentiation and natural selection were determined in order to study the polymorphic characteristic of PkMSP-1_42_.

**Results:**

A high level of genetic diversity (Hd = 0.970 ± 0.007; л = 0.01079 ± 0.00033) was observed among the 83 *P. knowlesi* samples, confirming the extensive genetic polymorphism exhibited among the *P. knowlesi* population found in Malaysia. A total of 18 distinct haplotypes with 17 amino acid changes were identified, whereby 15 were new haplotypes. High population differentiation values were observed within samples from Peninsular Malaysia and Malaysian Borneo. The 42 kDa fragments of *P. knowlesi* from Malaysian Borneo were found to be acting on balancing selection whilst purifying selection was suggested to act on isolates from Peninsular Malaysia. The separation of PkMSP-1_42_ haplotypes into two main groups based on geographical separation has further supported the existence of two distinct *P. knowlesi* lineages.

**Conclusions:**

A high level of genetic diversity was observed among PkMSP-1_42_ in Malaysia, whereby most of the polymorphisms were found within the 33 kDa region. Taken together, these data will be useful in order to understand the nature of *P. knowlesi* population in Malaysia as well as the design and development of a MSP-1_42_ based knowlesi malaria vaccine.

**Electronic supplementary material:**

The online version of this article (10.1186/s13071-018-3234-5) contains supplementary material, which is available to authorized users.

## Background

The scale-up of malaria control interventions has resulted in a substantial decline in global malaria morbidity and mortality. Despite this achievement, malaria remains a serious global health burden, resulting in 216 million cases annually and nearly half of the world’s population are at risk of malaria. The annual malaria-associated mortality reached 44,500 cases, primarily in children under five [1].

*Plasmodium knowlesi*, a zoonotic malaria parasite that is commonly found in long-tailed, pig-tailed and banded leaf macaques [2, 3], has recently been recognized as the fifth malaria-causing species in humans [4, 5]. Malaysia has achieved great success in controlling malaria over recent decades, in particular with marked reductions in the incidences of *Plasmodium falciparum* and *Plasmodium vivax* [6]. However, with increasing number of reported human knowlesi infections, *P. knowlesi* is now the predominant species occurring in this country, particularly in the state of Sabah, comprising 62% of cases in 2013 [7, 8].

Although the global reported prevalence of human infection with *P. knowles*i is considerably less than that caused by *P. falciparum* and *P. vivax* [8], the overall human burden of *P. knowlesi* infection may be substantially underestimated due to the use of routine microscopy which might lead to misidentification as other human malaria species [9]. Furthermore, the increasing overlap between macaque, human and vector habitats pose a major challenge for malaria control and elimination programmes [10]. Coupled with recent reports of severe and fatal consequences of knowlesi malaria in humans [11–14], this evidence further highlights the public health importance of this simian parasite, particularly in Malaysia.

Among the polymorphic loci of *Plasmodium*, merozoite surface protein-1 (MSP-1) has been widely used to study genetic diversity and is a prime vaccine candidate in clinical trials for many years [15]. Merozoite surface protein-1 (MSP-1) is a high molecular mass protein found on the surface of the blood stage of the parasite; it plays a key role during erythrocyte invasion [16, 17]. This 190 kDa precursor undergoes two steps of proteolytic processing during merozoite maturation. First, it is cleaved into four major fragments of 83, 30, 38 and 42 kDa, which remain on the merozoite surface as a glycosylphosphatidylinositol-anchored complex. Before erythrocyte invasion, the MSP-1_42_ fragment undergoes a second cleavage, resulting in the generation of the 33 and 19 kDa (MSP-1_33_ and MSP-1_19_) fragments, where the latter remains on the surface as the merozoite, which enters the erythrocyte [18, 19].

This 42 kDa fragment of MSP-1 is a promising vaccine candidate due to its high immunogenicity [20]. Several studies have reported that antibodies directed against the 42 and 19 kDa fragments of MSP-1 (MSP-1_42_ and MSP-1_19_) can interrupt merozoite invasion *in vitro* [21, 22]. However, extensive genetic polymorphism has also been reported in MSP-1_42_ of *P. falciparum* [23] and *P. vivax* [24, 25] among global isolates and this remains a major obstacle hampering the development of an effective malaria vaccine.

The genetic variation in the central repeat region of MSP-1 of *P. falciparum* [26–28] and *P. vivax* [24, 25] have been relatively well studied, but very little is known about the genetic diversity in this 42 kDa fragment of MSP-1 gene within *P. knowlesi* (PkMSP-1_42_). A pilot study of the sequence variation in *P. knowlesi* MSP-1_42_ has been previously described based on 12 clinical samples collected from hospitals in two states of Malaysia, i.e. Selangor and Sabah [29]. Nevertheless, small sample sizes can lead to significant errors in estimating the genetic diversity of the species. Therefore it is vital to include more samples in order to accurately characterize the genetic diversity, understand the parasite population history and, consequently, to assess the impact of elimination interventions [30].

The present study is an expansion from our previous work to further understand the genetic diversity, natural selection and haplotype groups of the gene fragment coding PkMSP-1_42_, whereby a larger sample size of *P. knowlesi* isolates (83 isolates) covering more states of Malaysia (eight states) are being included. The level of genetic diversity of *P. knowlesi* population in Malaysia may provide insight into trends in parasite transmission and be useful for the design and development of an MSP-1_42_ based knowlesi malaria vaccine.

## Methods

### Blood samples and geographical origin

**Fig. 1:**
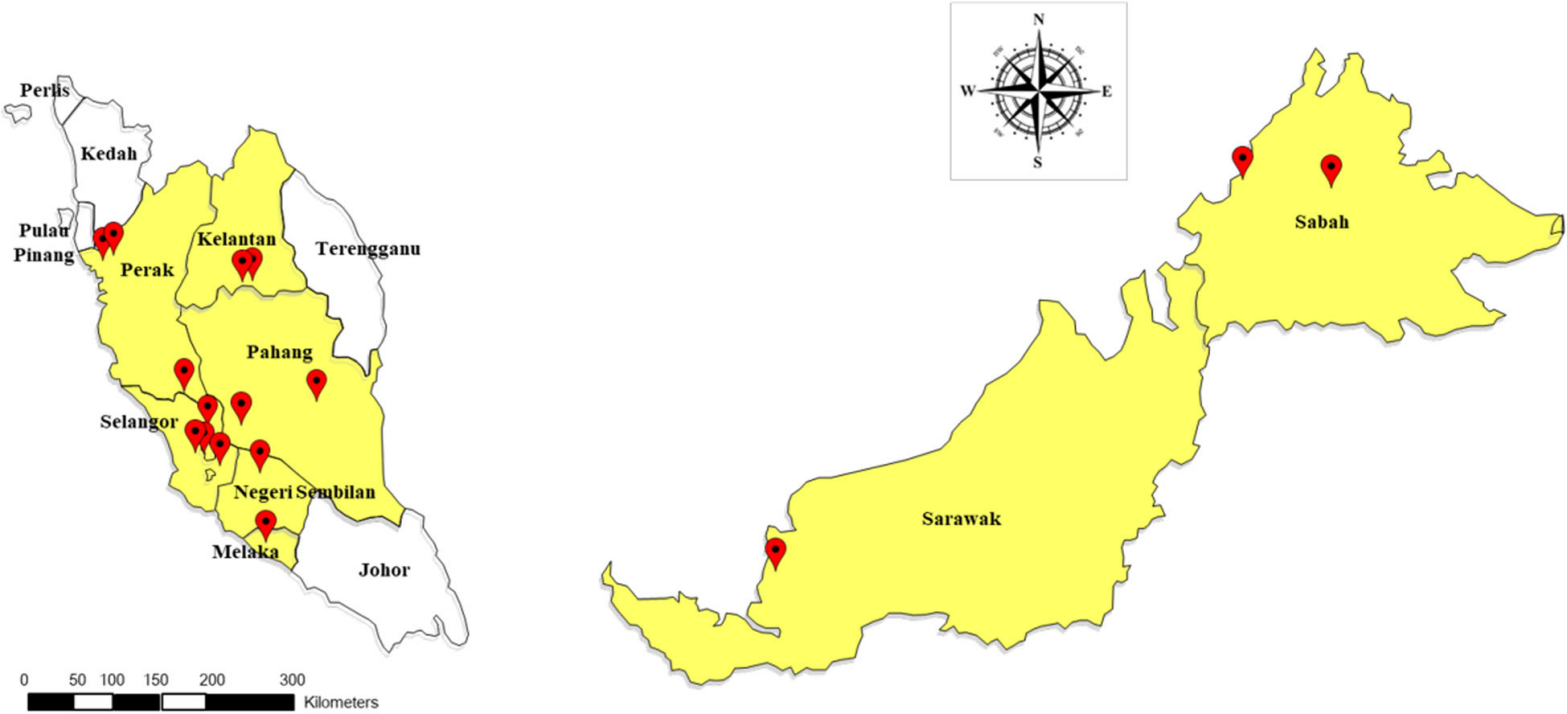
Geographical origin of samples used in this study

A total of 645 blood samples, collected from different states of Malaysia between 2011 and 2014, were screened *via* nested PCR for human *Plasmodium* species (i.e. *P. falciparum*, *P. vivax*, *P. malariae*, *P. ovale* and *P. knowlesi*) as previously described [5, 31]*.* Of these, 83 samples were found positive with single infection of *P. knowlesi* (Table 1), consisting of 43 clinical samples of *P. knowlesi* collected from health clinics and hospitals located in Selangor and Sabah, while 40 were *P. knowlesi* community samples collected among indigenous populations of various sub-tribes from 10 villages of six other states of Malaysia. The locations of the collection sites are shown in Fig 1.

**Table 1:** Distribution of *P. knowlesi* positive samples according to state in Malaysia (*N* = 83)

State	District	Village/ Hospital/ Health clinic	*n*
Peninsular Malaysia			
Perak	Slim River	Kg Sungai Bil	15
	Tapah	Kg Batu 7 1/2	4
		Kg Batu 8	7
Selangor	Hulu Selangor	Hospital Kuala Kubu Bharu	15
	Gombak	Hospital Selayang	4
	Petaling	Hospital Sungai Buloh	1
	Hulu Langat	Hospital Kajang	1
Pahang	Pekan	Kg Chini	1
	Lanchang	Kg Kuala Gandah	1
Melaka	Alor Gajah	Kg Bukit Sebang	1
Negeri Sembilan	Jelebu	Kg Ulu Kelaka	2
Kelantan	Gua Musang	Kg Kuala Lah	3
		Kg Aring 5	3
Malaysian Borneo			
Sabah	Kota Kinabalu	Hospital Queen Elizabeth II	12
	Beluran	Telupid Health Clinic	10
Sarawak	Sarikei	Pakan	3

### DNA extraction, PCR amplification and sequencing of PkMSP-1_42_

Whole genomic DNA was extracted from 200 μl of collected blood samples using a commercially available QIAamp DNA Blood Mini Kit (Qiagen, Hilden, Germany), according to the manufacturer’s instructions and stored at -20 °C until further use. PCR amplification of PkMSP-1_42_ was performed in a 50 μl reaction volume consisting of 5 μl of DNA template, 1× PCR buffer (Promega, Madison, USA), 0.3 mM dNTPs (Promega, Madison, USA), 3 mM MgCl_2_ (Promega, Madison, USA), 2.0 U of Taq DNA polymerase (Promega, Madison, USA) and 0.4 μM forward and reverse primers (PkMSP1-42F: 5'-TCT ACC CCT GTT CGG CAA TG-3' and PkMSP1-42R: 5'-CCA TGG AAA GGA AGA AAA TCA ACA-3') based on the previously described region [20]. Thermal condition was as follows: an initial cycle of activation at 95 °C for 5 min, followed by 35 cycles of denaturation at 94 °C for 1 min, annealing at 59 °C for 1 min and extension at 72 °C for 1 min, with a final extension of 72 °C for 10 min. The amplified products were examined in agarose gels at 1.5 % and then subjected to direct, automated sequencing (BigDye Terminator v.3.1 chemistry, Applied Biosystems, Foster City, USA). The quality of electropherograms with the forward and reverse nucleotide sequences was verified manually using Geneious v.9.0.4 software [32]. Sequence electropherograms were also carefully inspected for the presence of multiple sequence types.

### Sequence and phylogenetic analysis

Nucleotide and deduced amino acid sequences were aligned and analyzed, using Geneious v.9.0.4 software [32] with the reference sequence encoded by the H strain of *P. knowlesi* (GenBank: XM_002258546). Phylogenetic trees were constructed using the neighbor-joining and maximum likelihood methods as described in MEGA7 [33]. Bootstrap replicates of 1000 were used to test the robustness of the trees. All newly generated sequences were deposited in the GenBank database (MH796675-MH796757) (Additional file 1: Table S1).

### DNA sequence polymorphism analysis

DNA sequence polymorphism analysis was performed on the 83 PkMSP-1_42_ sequences. DnaSP v.5.10.01 [34] was used to calculate the numbers of segregating sites (S) and haplotypes (H) as well as haplotype diversity (Hd), nucleotide diversity (л) and average number of pairwise nucleotide differences within the population (K). The numbers of synonymous nucleotide substitutions per synonymous site (dS), the number of non-synonymous substitutions per non-synonymous site (dN), and the difference between the non-synonymous and synonymous substitutions (dN-dS) were estimated using Nei & Gojobori’s method [35] with the Jukes and Cantor (JC) correction to detect evidence of natural selection in MEGA7. In brief, an excess of dN relative to dS is a clear signal of positive selection. Conversely, a lack of dN relative to dS suggests a negative or purifying selection. Tajima’s D [36] and Fu and Li’s D and F test [37] were further applied using DnaSP version 5.10.01 to evaluate the neutral model of molecular evolution. A significantly positive value of Tajima’s D indicates balancing selection, whereas a negative value suggests negative selection or population size expansion after a recent bottleneck. Wright’s fixation index (F_ST_) was used to measure genetic differentiation between the PkMSP-1_42_ fragments of Peninsular Malaysia and Malaysian Borneo isolates [38]. F_ST_ values between populations were calculated using DnaSP v.5.10.01 based on the differences in allele frequencies. They are interpreted as no or low (0–0.05), moderate (0.05–0.15) and high (0.15–0.25) genetic differentiation.

## Results

### Genetic polymorphism and amino acid changes of PkMSP-1_42_

In this study, the region corresponding to PkMSP-1_42_ was successfully amplified from all 83 *P. knowlesi* Malaysia samples (58 from Peninsular Malaysia, 25 from Malaysian Borneo). This fragment of 993 bp in size contained a region coding a protein sequence of 331 amino acids. Using the *P. knowlesi* H strain sequence published in GenBank (XM_002258546) as a reference, 61 segregating sites were identified. Singleton sites were found to be lower in frequency (12/61) than the parsimony-informative sites (having a minimum of two nucleotides each present at least twice) (49/61). Of these polymorphic sites, 47 were dimorphic and two were trimophic changes (Additional file 2: Figure S1).

**Fig. 2:**
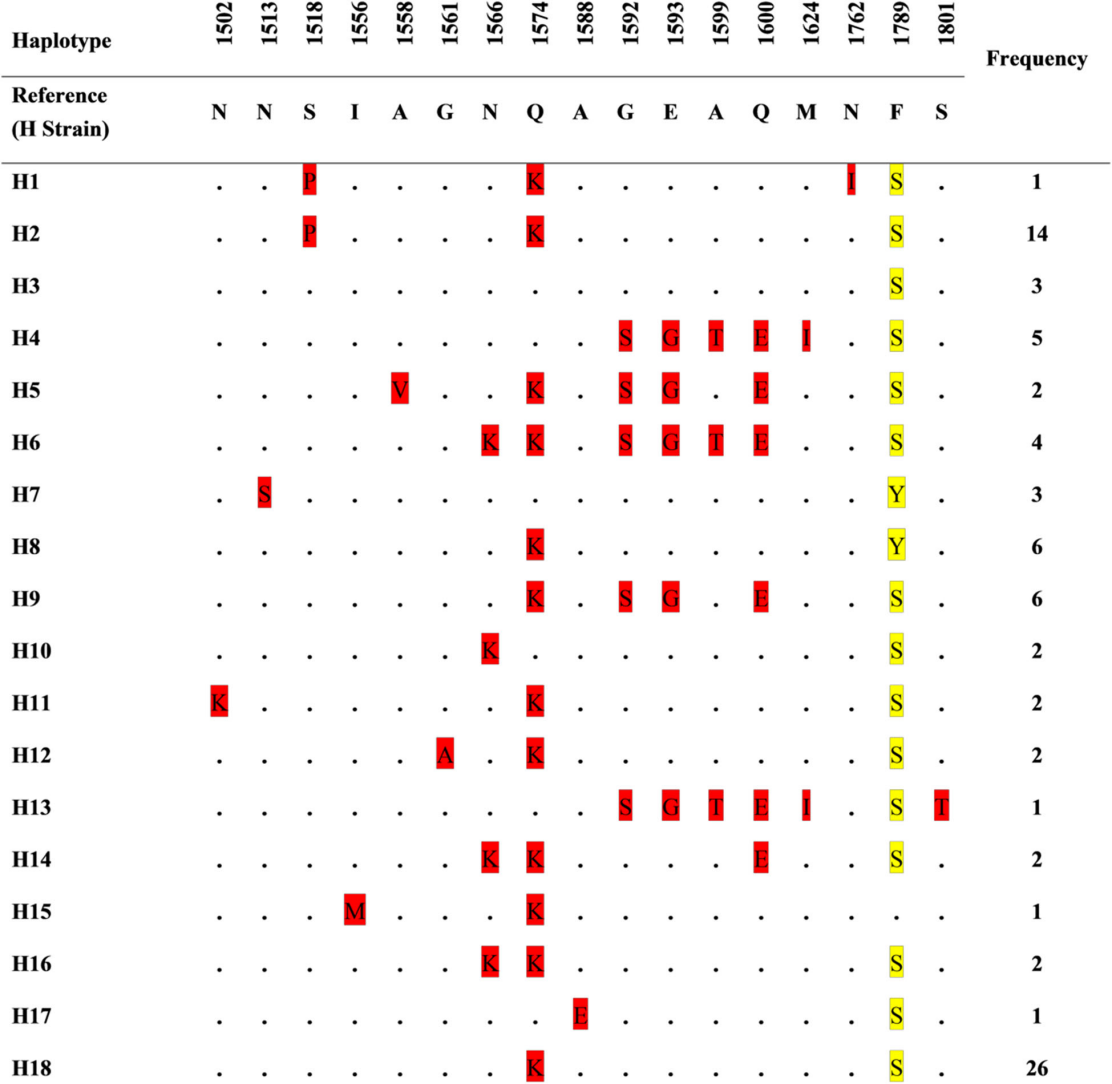
Amino acid sequence polymorphism in PkMSP-1_42_. Dimorphic and trimorphic amino acid changes are shaded in red and yellow, respectively. The total numbers of sequences for each haplotype are listed in the right panel

Amino acid changes at 17 positions were identified as compared to the reference H strain sequence. Of these, 16 were dimorphic mutations with a change into two amino acid type, while one showed trimorphic mutations with change in three amino acid types (F1789S/Y) (Fig. 2). Eighteen haplotypes were deduced from the amino acid sequences with haplotype H18 having the highest frequency (26/83, 31.3%). The distribution of PkMSP-1_42_ amino acid haplotypes in Peninsular Malaysia and Malaysian Borneo is shown in Table 2.

**Table 2:** Number of isolates for each of the PkMSP-1_42_ amino acid haplotypes in the two main study areas of Malaysia (*N* = 83)

Amino acid haplotypes	Study area		Total
	Peninsular Malaysia	Malaysian Borneo	
H1	1		1
H2	14		14
H3		3	3
H4		5	5
H5		2	2
H6		4	4
H7	3		3
H8	6		6
H9		6	6
H10	2		2
H11	1	1	2
H12	2		2
H13		1	1
H14		2	2
H15	1		1
H16	1	1	2
H17	1		1
H18	26		26
Total	58	25	83

### Nucleotide diversity and natural selection

DNA sequence polymorphisms analyses were performed to determine the nucleotide diversity and genetic differentiation at PkMSP-1_42_ region as well as for its 33 and 19 kDa fragments among the Malaysian *P. knowlesi* samples. The average number of pairwise nucleotide differences (K) for entire MSP-1_42_ region of all 83 samples was found to be 10.7338, while the overall haplotype diversity (Hd) and nucleotide diversity (л) were 0.970 ± 0.007 and 0.01079 ± 0.00033, respectively. The Malaysian Borneo PkMSP-1_42_ was noted to have slightly higher diversity (π = 0.01024 ± 0.00061) than the Peninsular Malaysia PkMSP-1_42_ (π = 0.009119 ± 0.00031). Analysis of the genetic diversity of the 33 and 19 kDa fragments of the samples from the entire population, revealed that PkMSP-1_33_ is more divergent as compared to the PkMSP-1_19_ fragment, suggesting that the nucleotide diversity was predominantly concentrated in PkMSP-1_33_ (Table 3). The overall haplotype diversity (Hd) and nucleotide diversity (π) for PkMSP-1_33_ was 0.963 ± 0.008 and 0.01010 ± 0.00044, respectively.

**Table 3:** Estimates of nucleotide diversity, natural selection and neutrality tests of PkMSP1-_42_ (*N* = 83)

Domain	S	л (SD)	*K*	Hd (SD)	dS	dN	dN-dS (SE)	Z-test (*P*-value)			Tajima’s D test	Fu & Li’s test	
								dN = dS	dN > dS	dN < dS		D	F
42 kDa													
Entire (*n* = 83)	61	0.01079 (0.00033)	10.7338	0.970 (0.007)	0.030	0.004	-0.026 (0.006)	<0.0001	1.000	<0.0001	-0.49252	-0.03809	-0.26440
Peninsular Malaysia (*n* = 58)	51	0.009119 (0.00031)	9.14156	0.956 (0.014)	0.029	0.002	-0.027 (0.006)	<0.0001	1.000	<0.0001	-0.68468	-1.11074	-1.13645
Malaysian Borneo (*n* = 25)	31	0.01024 (0.00061)	10.18667	0.910 (0.030)	0.035	0.004	-0.031 (0.008)	<0.0001	1.000	<0.0001	0.75884	0.74035	0.87508
33 kDa													
Entire (*n* = 83)	46	0.01010 (0.00044)	7.89891	0.963 (0.008)	0.026	0.004	-0.022 (0.006)	<0.0001	1.000	<0.0001	-0.52113	0.55872	0.15928
Peninsular Malaysia (*n* = 58)	37	0.00758 (0.00037)	5.92922	0.946 (0.015)	0.032	0.002	-0.030 (0.008)	<0.0001	1.000	<0.0001	-0.92293	-1.07888	-1.21884
Malaysian Borneo (*n* = 25)	25	0.01082 (0.00062)	8.46000	0.893 (0.034)	0.034	0.005	-0.029 (0.009)	0.001	1.000	0.001	0.84595	0.96956	1.09115
19 kDa													
Entire (*n* = 83)	15	0.00779 (0.00064)	2.83485	0.916 (0.011)	0.046	0.002	-0.044 (0.016)	0.001	1.000	0.004	-0.33245	-1.37274	-1.18780
Peninsular Malaysia (*n* = 58)	14	0.00708 (0.00067)	3.21234	0.906 (0.019)	0.068	0.002	-0.066 (0.024)	0.003	1.000	0.001	-0.02596	-0.81452	-0.64299
Malaysian Borneo (*n* = 25)	6	0.00811 (0.00117)	1.72667	0.773 (0.048)	0.038	0.000	-0.038 (0.018)	0.001	1.000	<0.0001	-0.25913	-0.25602	-0.12303

*Abbreviations*: *N* number of isolates, *S* number of segregating sites, *K* average number of pairwise nucleotide differences, *Hd* haplotype diversity, *л* observed average pairwise nucleotide diversity, *dS* nucleotide diversity of synonymous mutation per synonymous site, *dN* nucleotide diversity of non-synonymous mutation per non-synonymous site; *dN, dS* the difference of dN and dS with their standard deviation estimated by bootstrap with 1000 pseudoreplicates, *SD* standard deviation

The genetic differentiation of PkMSP-1_42_ as well as PkMSP-1_33_ and PkMSP-1_19_ fragments among the samples from Peninsular Malaysia and Malaysian Borneo were compared using Wright’s fixation index (F_ST_). The findings showed high genetic differentiation within the *P. knowlesi* populations originating from Peninsular Malaysia and Malaysian Borneo in both PkMSP-1_42_ and PkMSP-1_33_ fragments, respectively (F_ST_: 0.23677, *P* < 0.001; 0.28257, *P* < 0.001). However, moderate genetic differentiation was observed in PkMSP-1_19_ fragments between Peninsular Malaysia and Malaysian Borneo (F_ST_: 0.05729, *P* < 0.001) (Table 4).

**Table 4:** F_ST_ indices for PkMSP-1_42_, PkMSP-1_33_ and PkMSP-1_19_ from Peninsular Malaysia and Malaysian Borneo

	PkMSP-1_42_	PkMSP-1_33_	PkMSP-1_19_
	Malaysian Borneo		
Peninsular Malaysia	0.23677*	0.28257*	0.05729*

*P < 0.001

In order to examine whether natural selection contributed to the diversity observed in PkMSP-1_42_, we further analyzed the average difference of dN-dS for all PkMSP-1_42_ sequences. The significant excess of synonymous substitutions (dN-dS = -0.026, *P* < 0.05) of PkMSP-1_42_ for all 83 samples as well as the samples from Peninsular Malaysia (dN-dS = -0.027, *P* < 0.05) and Malaysian Borneo (dN-dS = -0.031, *P* < 0.05), respectively, suggested a purifying selection for MSP-1_42_ fragment of *P. knowlesi* populations from both Peninsular Malaysia and Malaysian Borneo areas. The negative values of Tajima’s D test (-0.68468) as well as Fu and Li’s D (-1.11074) and F (-1.13645) further supported the proposal for a purifying selection pressure of the PkMSP-1_42_ region for samples from Peninsular Malaysia. On the other hand, the high positive values of Tajima’s D (0.75884), Fu and Li’s D (0.74035) and F (0.87508) found among PkMSP-1_42_ of Malaysian Borneo samples instead suggested balancing selection acts on the fragment in the samples collected from this region (Table 3). When analysis was done for 33 and 19 kDa fragments separately, similar findings were observed in the PkMSP-1_33_ fragment whereby negative purifying selection was suggested to be acting among Peninsular Malaysia samples. High positive values of Tajima’s D and Fu and Li’s D and F among Malaysian Borneo samples suggesting that PkMSP-1_33_ fragments from this region may be under balancing selection pressure. On the other hand, purifying selection is most likely to be acting at the fragment of PkMSP-1_19_ among both Peninsular Malaysia and Malaysian Borneo samples (Table 3).

### Phylogenetic analysis

**Fig. 3:**
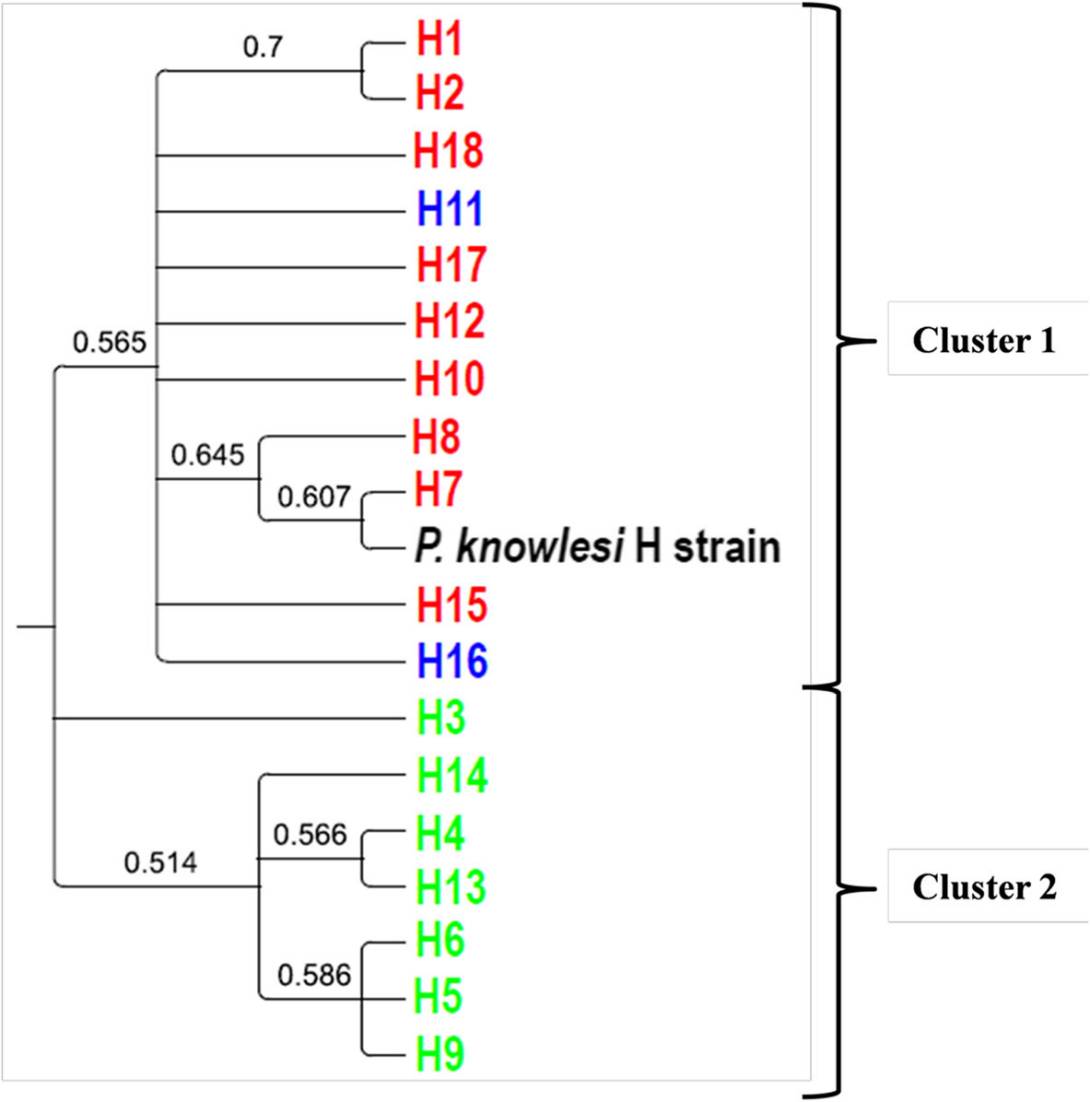
Phylogenetic tree of PkMSP-1_42_ haplotypes. The neighbor-joining method was used to construct the tree. Numbers at nodes indicate percentage support of 1000 bootstrap replicates; only bootstrap values above 50% are shown. Haplotypes from Peninsular Malaysia and Malaysian Borneo are indicated in red and light green, respectively, while the shared haplotypes are indicated in blue

**Fig. 4:**
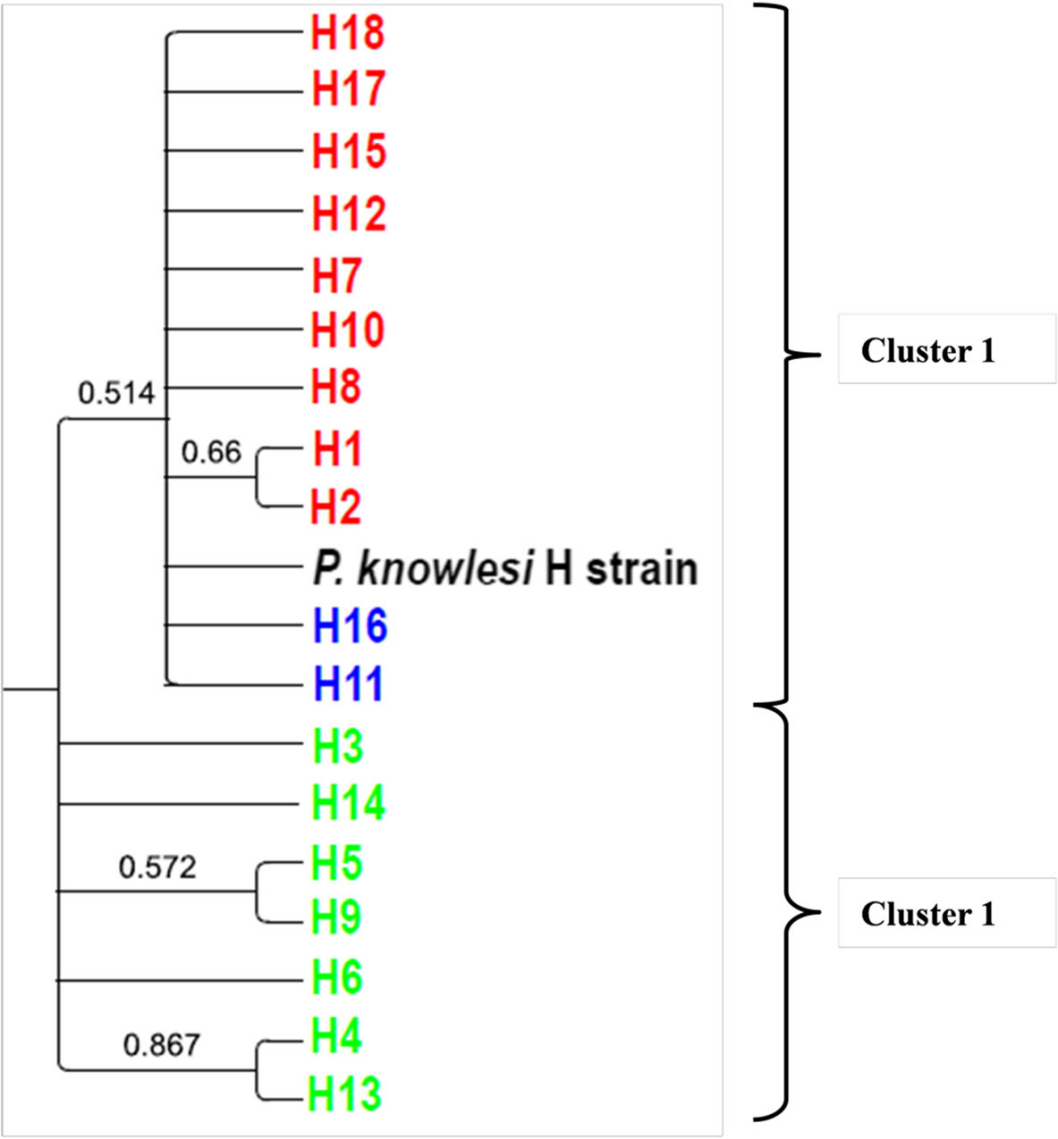
Phylogenetic tree of PkMSP-1_42_ haplotypes. The maximum likelihood method was used to construct the tree. Numbers at nodes indicate percentage support of 1000 bootstrap replicates; only bootstrap values above 50% are shown. Haplotypes from Peninsular Malaysia and Malaysian Borneo are indicated in red and light green, respectively, while the shared haplotypes are indicated in blue

**Fig. 5:**
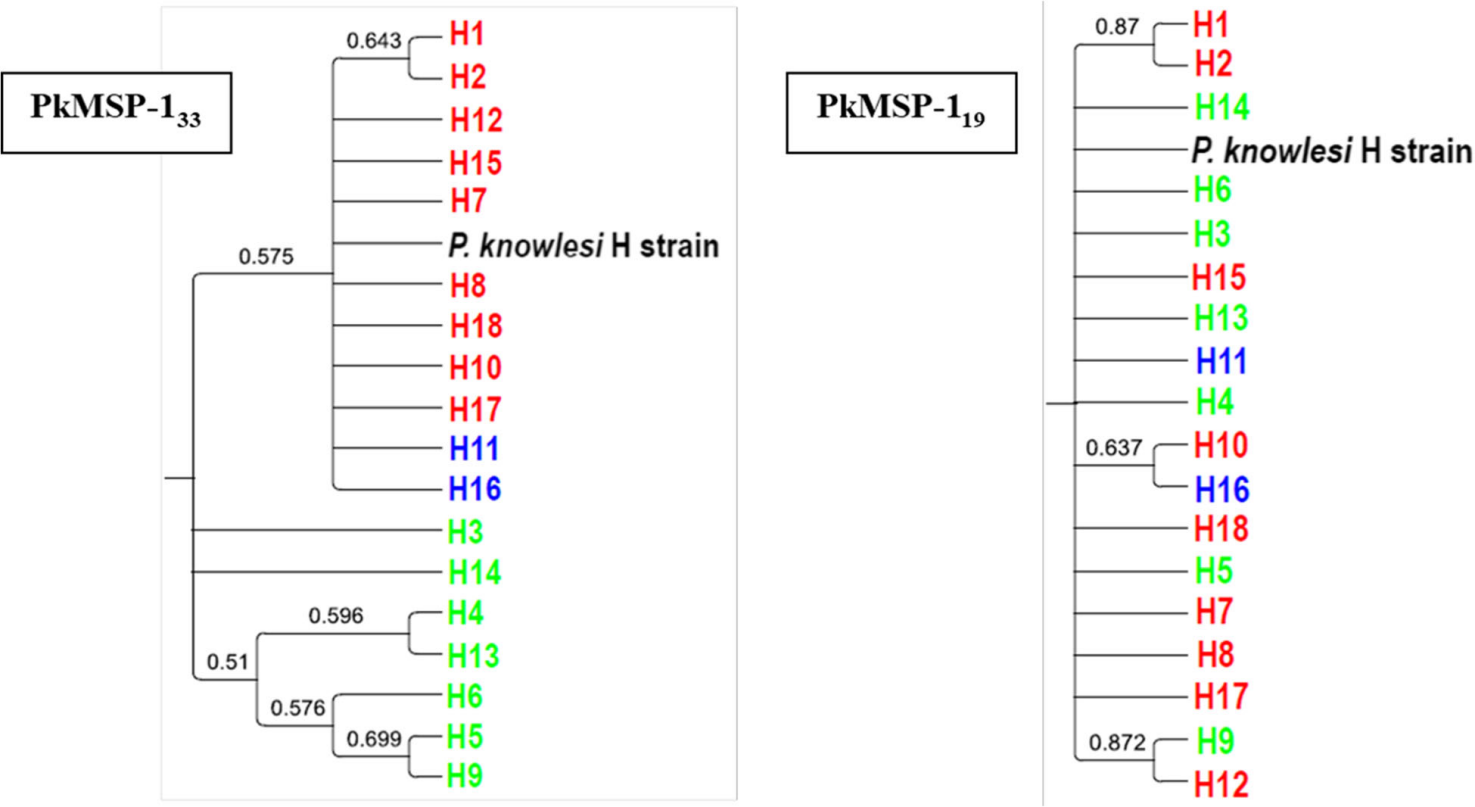
Phylogenetic tree of PkMSP-1_33_ and PkMSP-1_19_ haplotypes. The neighbor-joining method was used to construct the tree. Numbers at nodes indicating percentage support of 1000 bootstrap replicates; only bootstrap values above 50% are shown. Haplotypes from Peninsular Malaysia and Malaysian Borneo are indicated in red and light green, respectively, while the shared haplotypes are indicated in blue

Phylogenetic trees of the 18 PkMSP-1_42_ amino acid haplotypes were constructed using both neighbor-joining and maximum likelihood methods. Both methods produced phylogenetic trees of similar topology and analysis revealed that the Malaysian PkMSP-1_42_ haplotypes were clustered into two main clades. Most of the samples collected from Malaysian Borneo formed one cluster while *P. knowlesi* samples from Peninsular Malaysia and two shared haplotypes formed another cluster with a laboratory line, the H strain, which also originated from Peninsular Malaysia (Figs. 3, 4). Another neighbor-joining tree was constructed for both PkMSP-1_33_ and PkMSP-1_19_ fragments to determine if they contributed to the haplotype clustering. We found that the phylogenetic tree of PkMSP-1_33_ displayed a similar clustering pattern to the tree constructed using PkMSP-1_42_, suggesting the 33 kDa fragment is playing the role of haplotype clustering as compared to PkMSP-1_19_ (Fig. 5).

## Discussion

The MSP-1_42_ is one of the most outstanding malarial vaccine antigens, which is currently at an advanced stage of clinical evaluation [39–41]. However, its extensive polymorphic nature suggests that continuous survey of the genetic polymorphism from a wide range of field isolates is necessary. To date, a considerable amount of studies on MSP-1_42_ have been carried out on *P. falciparum* [42, 43] and *P. vivax* [24, 25, 44] but there is a paucity of information on the structure, function or genetic variation in MSP-1 in *P. knowlesi*. Recent studies by Cheong et al. [20, 45] demonstrated the high immunogenicity of MSP-1_42_ and its ability to elicit protective immunity in *P. knowlesi*. This suggests that PkMSP-1_42_ may serve as a candidate for malaria vaccine design. However, further evaluation is needed to validate its potential and limitations.

Previously, we conducted a pilot study with sample size of 12 *P. knowlesi* clinical isolates to determine the genetic diversity of PkMSP-1_42_ [29]. However, a larger sample size is important for precise estimation of genetic diversity parameters in order to characterize the parasite population [30]. Hence, we expanded our work using the same approach, but with a larger sample size covering more states of Malaysia in order to study the genetic polymorphism and natural selection of MSP-1_42_ in Malaysian *P. knowlesi* samples at a broader scale. All 83 sequences of single *P. knowlesi* infections were classified into 18 distinct haplotypes with amino acid changes at 17 positions as compared to the reference H strain sequence. Most of the identified haplotypes were novel and have not been reported previously, except for haplotypes H2, H7 and H18 in our previous study [29]. Most of the amino acid substitutions were found concentrated in the PkMSP-1_33_ region whereby only two dimorphic changes (N1762I, S1801Y) and one trimophic change (F1789S/Y) were seen in PkMSP-1_19_. It is known that MSP-1_19_ is highly conserved, in all *Plasmodium* species including PkMSP-1_19_ of clinical isolates from Malaysia which has recently been reported [46].

The high level of genetic polymorphism observed in the present study (л = 0.0179) was in concordance with the result reported previously (л = 0.0132) [29], further confirming the extensiveness of genetic polymorphism among *P. knowlesi* population found in Malaysia. This finding is also in agreement with a previous analysis on MSP-1_42_ fragments reported in *P. knowlesi*-infected orangutan samples from Kalimantan, Indonesia (л = 0.013) [47] as well as other *P. knowlesi* functional genes, such as Pk-DBPαII (π = 0.013 ± 0.002) [48] and Pk-RAP-1 (π = 0.01298 ± 0.00091) [49]. However, this diversity was lower than the MSP-1_42_ fragment reported previously for both *P. falciparum* [23, 26, 28] and *P. vivax* [24, 25, 44]. With regards to non-human primate malaria, PkMSP-1_42_ was also found to be less diverse than MSP-1_42_ of *P. cynomolgi* and *P. inui* [47, 50]. The lower genetic diversity of *P. knowlesi* human infection as compared to other *Plasmodium* species may be related to the limited intensity of malaria transmission in which to date, human-to-human transmission has not been reported.

The rate of non-synonymous and synonymous mutations (dN-dS) is often used to evaluate the effect of natural selection on gene sequences. Most of the malaria surface antigens with relatively high polymorphism have been reported to be under positive-diversifying selection due to the accumulation of amino acid replacements that may hamper the ability of the host’s immune system to recognize the parasite [51]. However, in the present study, the significant negative values of dN-dS (-0.026) and Tajima’s D statistic (-0.49252) in the entire population of PkMSP-1_42_ sequences suggest that the MSP-1_42_ fragment in our *P. knowlesi* samples is under the influence of negative natural selection. This finding is in accordance with our previous study on 12 PkMSP-1_42_ human *P. knowlesi* sequences from Malaysia [29] as well as *P. knowlesi* from orangutan samples from Kalimantan, Indonesia [47].

When the PkMSP-1_42_ sequences of Peninsular Malaysia and Malaysian Borneo were further analysed as two different populations, some differences were observed whereby the Malaysian Borneo PkMSP-1_42_ was found to have slightly higher diversity (π = 0.01024 ± 0.00061) as compared to those from Peninsular Malaysia (π = 0.009119 ± 0.00031). Although significant negative values of dN-dS were seen on PkMSP-1_42_ as well as PkMSP-1_33_ and PkMSP-1_19_ of both Peninsular Malaysia and Malaysian Borneo populations, negative values of Tajima’s D postulated purifying selection were acting on 42 and 33 kDa fragments of *P. knowlesi* from Peninsular Malaysia, whereas positive values of Tajima’s D in PkMSP-1_42_ and PkMSP-1_33_ of Malaysian Borneo populations suggested they are under balancing selection pressure. This suggests that a bottleneck event may be happening among *P. knowlesi* in Peninsular Malaysia during the transition period of parasite transmission from macaque host to humans that drives population expansion or growth. On the other hand, the balancing selection pressure acting on *P. knowlesi* in Malaysian Borneo population suggests that the active expression of different alleles of the gene allow *P. knowlesi* to escape the human immune response, thus maintaining a high infection rate. Given that most of the human knowlesi infections were reported from Sabah and Sarawak, Malaysian Borneo [52], this might also be one of the reasons why the selection pressure acting on *P. knowlesi* sampled from respective Peninsular Malaysia and Malaysian Borneo populations in our study was different. Nevertheless, the low level of genetic polymorphism and negative purifying selection found among PkMSP-1_19_ in both Peninsular Malaysia and Malaysian Borneo populations are in agreement with previous studies [29, 46].

Furthermore, the high genetic differentiation using Wright’s fixation index (F_ST_) was seen in both PkMSP-1_42_ and PkMSP-1_33_ within Peninsular Malaysia and Malaysian Borneo *P. knowlesi* populations. This might be due to the geographical separation of Peninsular Malaysia and Malaysian Borneo by the South China Sea. These results are similar to previous findings at the genomic level as well as functional genes [46, 53, 54]. On the other hand, the moderate genetic differentiation of PkMSP-1_19_ seen within Peninsular Malaysia and Malaysian Borneo populations further confirmed the theory of the 19 kDa fragment being a highly conserved region which is evolving towards a fixation stage.

Unlike a previous study in which no evidence of geographical clustering was seen among the PkMSP-1_42_ [29], the phylogenetic tree analyses of the present study involving a larger sample size revealed that *P. knowlesi* MSP-1_42_ haplotypes were clustered into two main groups: one in which samples from Peninsular Malaysia clustered with the laboratory H strain, and the other comprising most of the samples typed from Malaysian Borneo. This is in agreement with previous reports on other *P. knowlesi* functional proteins such as the Duffy binding protein (PkDBPαII) [48], Pknbpxa [55], PkAMA-1 domain [56] and PkMSP-3 [57] which reported bifurcation of haplotypes, indicating dimorphism of the genes. Recent microsatellite genotyping of *P. knowlesi* found in humans and macaques have also highlighted the presence of two divergent *P. knowlesi* populations which have been associated with two natural macaque reservoir host species, the long tailed (*Macaca fascicularis*) and the pig tailed macaque (*Macaca nemestrina*) [58].

It is important to characterize genetic diversity in order to understand parasite biology and disease pathogenesis, to evaluate the direct effects of diversity on clinical disease, as well as to develop an effective malaria vaccine [59]. Several studies have associated the genetic diversity of *P. falciparum* with the clinical outcome of a malaria infection comparing different genotypic determinants in mild and severe cases [60–62]. However, the limitation of our study is that we could not obtain the clinical status of all our 83 *P. knowlesi*-infected samples; therefore, we were not able to determine whether the severity of malaria episode was associated with a particular genotype of PkMSP-1_42_.

## Conclusions

The present study provides an in-depth analysis of the genetic diversity and natural selection of the PkMSP-1_42_ gene among Malaysian samples. PkMSP-1_42_ showed polymorphic characteristics that resulted in 18 distinct haplotypes. Synonymous and non-synonymous mutation analysis indicated negative purifying selection of the gene on the overall *P. knowlesi* Malaysia population. The separation of PkMSP-1_42_ haplotypes into two main groups has further supported the existence of two distinct *P. knowlesi* lineages. Most polymorphisms were found in the 33 kDa fragment while the 19 kDa fragment was relatively conserved, highlighting that future studies should focus more in PkMSP-1_33_ as this region appears to be more informative for the development of a knowlesi malaria vaccine.

## Additional files

Additional file 1: **Table S1.** Study samples and origin. (DOCX 20 kb)

Additional file 2: **Figure S1.** Nucleotide variants of 38 haplotypes of *P. knowlesi* MSP1-_42_. Identical nucleotides are marked as dots, while polymorphic sites are shaded in light green (two variants) and light blue (three variants), respectively. (ZIP 699 kb)

## Abbreviations

dNTPs: Deoxynucleotide triphosphates
PCR: Polymerase chain reaction
