# Supplementary material for: Genetic polymorphism and natural selection in the C-terminal 42 kDa region of merozoite surface protein-1 (MSP-1) among Plasmodium knowlesi samples from Malaysia
Source: Parasit Vectors. 2018 Dec 5;11:626. doi: 10.1186/s13071-018-3234-5 (PMC6282282; doi:10.1186/s13071-018-3234-5)
Supplement: Supplementary file 1 — Table S1. Study samples and origin. (DOCX 20 kb) [file 13071_2018_3234_MOESM1_ESM.docx]

**Additional File 1**

Additional File 1, Table 1: Study samples and origin

| **No** | **Sample** | **Location** | **Haplotype** |
| --- | --- | --- | --- |
| 1 | MH796675 | Pakan, Sarikei, Sarawak | 3 |
| 2 | MH796676 | Pakan, Sarikei, Sarawak | 3 |
| 3 | MH796677 | Pakan, Sarikei, Sarawak | 3 |
| 4 | MH796678 | Hospital Queen Elizabeth, Kota Kinabalu, Sabah | 4 |
| 5 | MH796679 | Hospital Queen Elizabeth, Kota Kinabalu, Sabah | 4 |
| 6 | MH796680 | Hospital Queen Elizabeth, Kota Kinabalu, Sabah | 4 |
| 7 | MH796681 | Hospital Queen Elizabeth, Kota Kinabalu, Sabah | 4 |
| 8 | MH796682 | Hospital Queen Elizabeth, Kota Kinabalu, Sabah | 4 |
| 9 | MH796683 | Hospital Queen Elizabeth, Kota Kinabalu, Sabah | 5 |
| 10 | MH796684 | Hospital Queen Elizabeth, Kota Kinabalu, Sabah | 5 |
| 11 | MH796685 | Hospital Queen Elizabeth, Kota Kinabalu, Sabah | 11 |
| 12 | MH796686 | Hospital Queen Elizabeth, Kota Kinabalu, Sabah | 13 |
| 13 | MH796687 | Hospital Queen Elizabeth, Kota Kinabalu, Sabah | 14 |
| 14 | MH796688 | Hospital Queen Elizabeth, Kota Kinabalu, Sabah | 14 |
| 15 | MH796689 | Hospital Queen Elizabeth, Kota Kinabalu, Sabah | 16 |
| 16 | MH796690 | Telupid Health Clinic, Beluran, Sabah | 6 |
| 17 | MH796691 | Telupid Health Clinic, Beluran, Sabah | 6 |
| 18 | MH796692 | Telupid Health Clinic, Beluran, Sabah | 6 |
| 19 | MH796693 | Telupid Health Clinic, Beluran, Sabah | 6 |
| 20 | MH796694 | Telupid Health Clinic, Beluran, Sabah | 9 |
| 21 | MH796695 | Telupid Health Clinic, Beluran, Sabah | 9 |
| 22 | MH796696 | Telupid Health Clinic, Beluran, Sabah | 9 |
| 23 | MH796697 | Telupid Health Clinic, Beluran, Sabah | 9 |
| 24 | MH796698 | Telupid Health Clinic, Beluran, Sabah | 9 |
| 25 | MH796699 | Telupid Health Clinic, Beluran, Sabah | 9 |
| 26 | MH796700 | Kg Bukit Sebang, Alor Gajah, Melaka | 1 |
| 27 | MH796701 | Kg Chini, Pekan, Pahang | 11 |
| 28 | MH796702 | Kg Kuala Gandah, Lanchang, Pahang | 16 |
| 29 | MH796703 | Kg Ulu Kelaka, Jelebu, Negeri Sembilan | 12 |
| 30 | MH796704 | Kg Ulu Kelaka, Jelebu, Negeri Sembilan | 12 |
| 31 | MH796705 | Kg Kuala Lah, Gua Musang, Kelantan | 8 |
| 32 | MH796706 | Kg Kuala Lah, Gua Musang, Kelantan | 8 |
| 33 | MH796707 | Kg Kuala Lah, Gua Musang, Kelantan | 8 |
| 34 | MH796708 | Kg Aring 5, Gua Musang, Kelantan | 8 |
| 35 | MH796709 | Kg Aring 5, Gua Musang, Kelantan | 8 |
| 36 | MH796710 | Kg Aring 5, Gua Musang, Kelantan | 8 |
| 37 | MH796711 | Kg Sungai Bil, Slim River, Perak | 10 |
| 38 | MH796712 | Kg Sungai Bil, Slim River, Perak | 10 |
| 39 | MH796713 | Kg Sungai Bil, Slim River, Perak | 15 |
| 40 | MH796714 | Kg Sungai Bil, Slim River, Perak | 18 |
| 41 | MH796715 | Kg Sungai Bil, Slim River, Perak | 18 |
| 42 | MH796716 | Kg Sungai Bil, Slim River, Perak | 18 |
| 43 | MH796717 | Kg Sungai Bil, Slim River, Perak | 18 |
| 44 | MH796718 | Kg Sungai Bil, Slim River, Perak | 18 |
| 45 | MH796719 | Kg Sungai Bil, Slim River, Perak | 18 |
| 46 | MH796720 | Kg Sungai Bil, Slim River, Perak | 18 |
| 47 | MH796721 | Kg Sungai Bil, Slim River, Perak | 18 |
| 48 | MH796722 | Kg Sungai Bil, Slim River, Perak | 18 |
| 49 | MH796723 | Kg Sungai Bil, Slim River, Perak | 18 |
| 50 | MH796724 | Kg Sungai Bil, Slim River, Perak | 18 |
| 51 | MH796725 | Kg Batu 7 1/2, Tapah, Perak | 2 |
| 52 | MH796726 | Kg Batu 7 1/2, Tapah, Perak | 2 |
| 53 | MH796727 | Kg Batu 7 1/2, Tapah, Perak | 2 |
| 54 | MH796728 | Kg Batu 7 1/2, Tapah, Perak | 2 |
| 55 | MH796729 | Kg Batu 8, Tapah, Perak | 18 |
| 56 | MH796730 | Kg Batu 8, Tapah, Perak | 18 |
| 57 | MH796731 | Kg Batu 8, Tapah, Perak | 18 |
| 58 | MH796732 | Kg Batu 8, Tapah, Perak | 18 |
| 59 | MH796733 | Kg Batu 8, Tapah, Perak | 18 |
| 60 | MH796734 | Kg Batu 8, Tapah, Perak | 18 |
| 61 | MH796735 | Kg Batu 8, Tapah, Perak | 7 |
| 62 | MH796736 | Hospital Kuala Kubu Bahru, Hulu Selangor, Selangor | 18 |
| 63 | MH796737 | Hospital Kuala Kubu Bahru, Hulu Selangor, Selangor | 18 |
| 64 | MH796738 | Hospital Kuala Kubu Bahru, Hulu Selangor, Selangor | 18 |
| 65 | MH796739 | Hospital Kuala Kubu Bahru, Hulu Selangor, Selangor | 18 |
| 66 | MH796740 | Hospital Kuala Kubu Bahru, Hulu Selangor, Selangor | 18 |
| 67 | MH796741 | Hospital Kuala Kubu Bahru, Hulu Selangor, Selangor | 18 |
| 68 | MH796742 | Hospital Kuala Kubu Bahru, Hulu Selangor, Selangor | 2 |
| 69 | MH796743 | Hospital Kuala Kubu Bahru, Hulu Selangor, Selangor | 2 |
| 70 | MH796744 | Hospital Kuala Kubu Bahru, Hulu Selangor, Selangor | 18 |
| 71 | MH796745 | Hospital Kuala Kubu Bahru, Hulu Selangor, Selangor | 2 |
| 72 | MH796746 | Hospital Kuala Kubu Bahru, Hulu Selangor, Selangor | 2 |
| 73 | MH796747 | Hospital Kuala Kubu Bahru, Hulu Selangor, Selangor | 2 |
| 74 | MH796748 | Hospital Kuala Kubu Bahru, Hulu Selangor, Selangor | 2 |
| 75 | MH796749 | Hospital Kuala Kubu Bahru, Hulu Selangor, Selangor | 2 |
| 76 | MH796750 | Kg Sungai Bil, Slim River, Perak | 18 |
| 77 | MH796751 | Hospital Kuala Kubu Bahru, Hulu Selangor, Selangor | 2 |
| 78 | MH796752 | Hospital Selayang, Gombak, Selangor | 2 |
| 79 | MH796753 | Hospital Selayang, Gombak, Selangor | 7 |
| 80 | MH796754 | Hospital Selayang, Gombak, Selangor | 18 |
| 81 | MH796755 | Hospital Selayang, Gombak, Selangor | 7 |
| 82 | MH796756 | Hospital Sungai Buloh, Petaling, Selangor | 2 |
| 83 | MH796757 | Hospital Kajang, Hulu Langat, Selangor | 17 |
