# Supplementary figures and images for: Genetic polymorphism and natural selection in the C-terminal 42 kDa region of merozoite surface protein-1 (MSP-1) among Plasmodium knowlesi samples from Malaysia
Source: Parasit Vectors. 2018 Dec 5;11:626. doi: 10.1186/s13071-018-3234-5 (PMC6282282; doi:10.1186/s13071-018-3234-5)

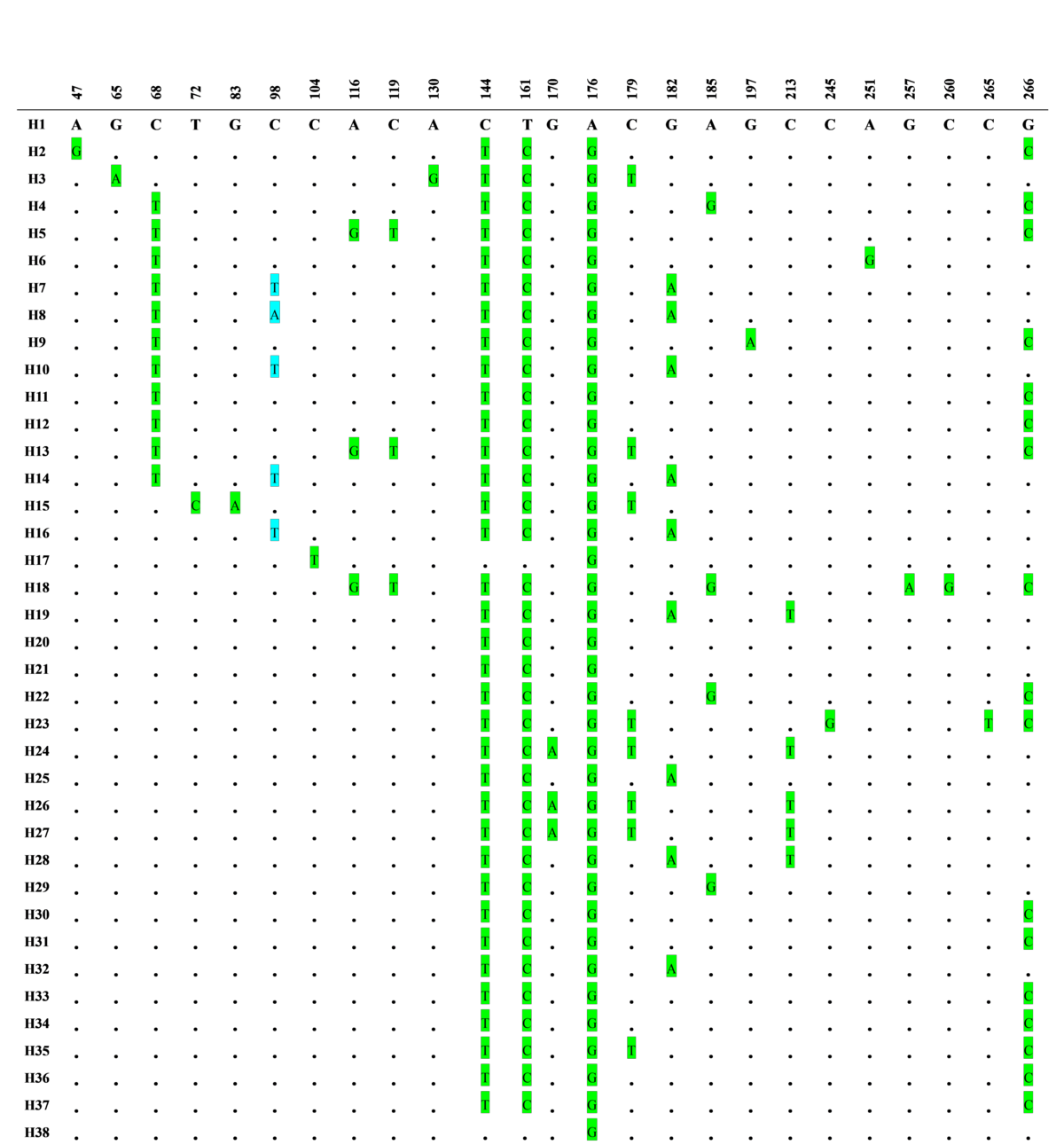

Supplement: Supplementary file 2 — Figure S1. Nucleotide variants of 38 haplotypes of P. knowlesi MSP1-42. Identical nucleotides are marked as dots, while polymorphic sites are shaded in light green (two variants) and light blue (three variants), respectively. (ZIP 699 kb) [file 13071_2018_3234_MOESM2_ESM.zip › Nan Jiun Yap_Additional File 2 Fig. S1_1.tiff]

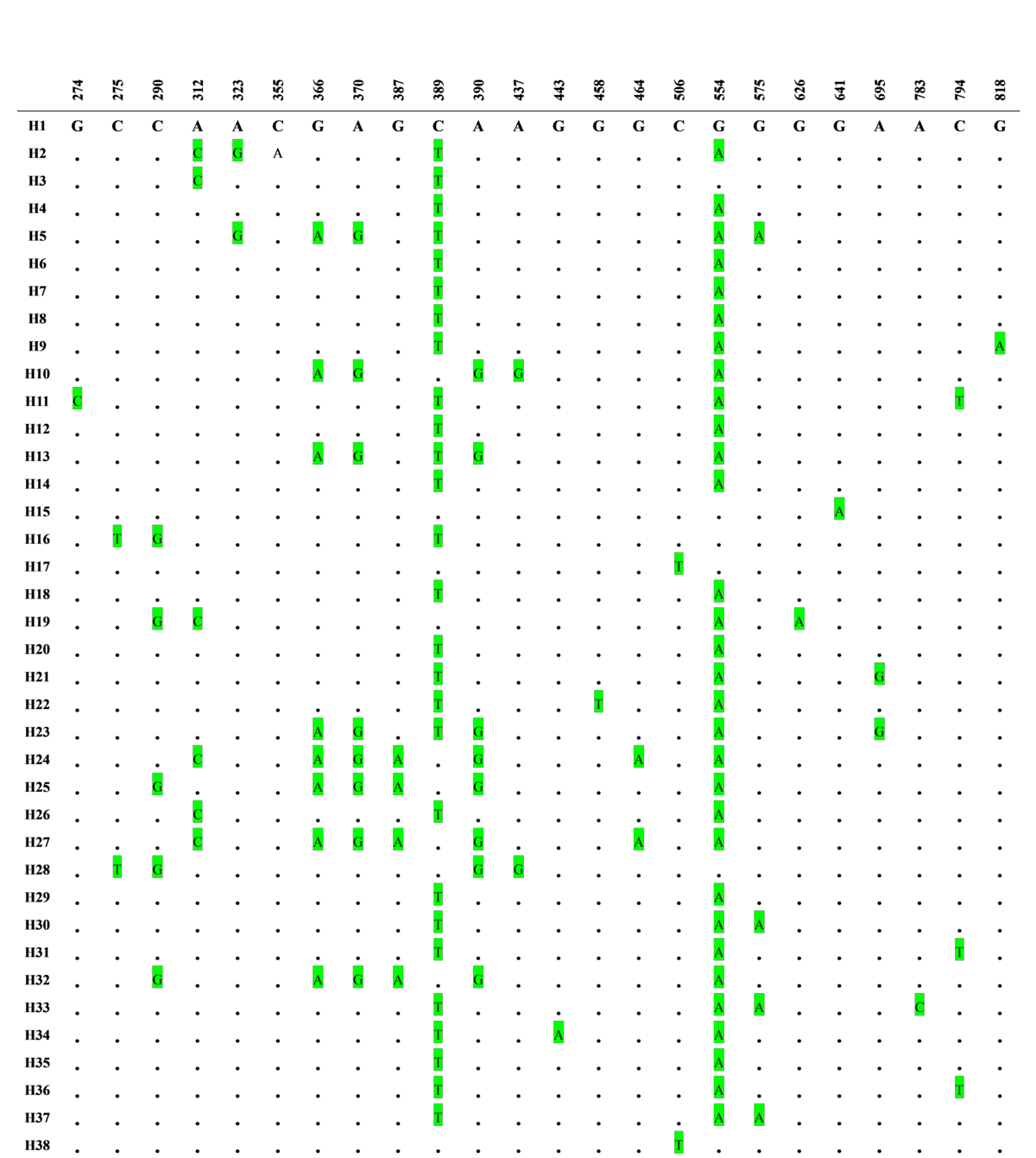

Supplement: Supplementary file 2 — Figure S1. Nucleotide variants of 38 haplotypes of P. knowlesi MSP1-42. Identical nucleotides are marked as dots, while polymorphic sites are shaded in light green (two variants) and light blue (three variants), respectively. (ZIP 699 kb) [file 13071_2018_3234_MOESM2_ESM.zip › Nan Jiun Yap_Additional File 2 Fig. S1_2.tiff]

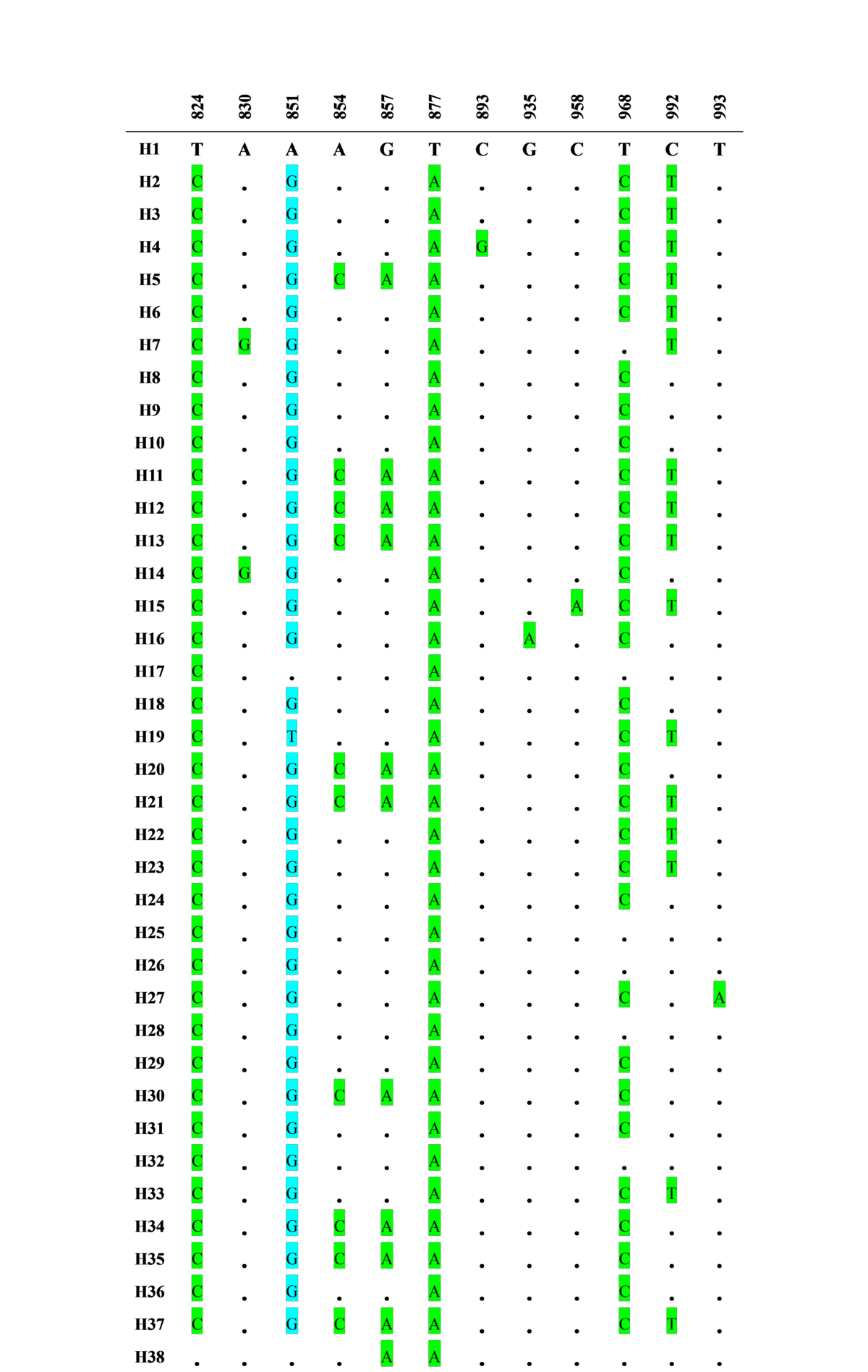

Supplement: Supplementary file 2 — Figure S1. Nucleotide variants of 38 haplotypes of P. knowlesi MSP1-42. Identical nucleotides are marked as dots, while polymorphic sites are shaded in light green (two variants) and light blue (three variants), respectively. (ZIP 699 kb) [file 13071_2018_3234_MOESM2_ESM.zip › Nan Jiun Yap_Additional File 2 Fig. S1_3.tiff]
